# Longitudinal Degradation of Pavement Marking Detectability for Mobile LiDAR Sensing Technology in Real-World Use

**DOI:** 10.3390/s23135815

**Published:** 2023-06-22

**Authors:** Byoung-Keon D. Park, James R. Sayer, André D. Clover, Matthew P. Reed

**Affiliations:** 1 University of Michigan Transportation Research Institute, University of Michigan, Ann Arbor, MI 48109, USA; jimsayer@umich.edu (J.R.S.); mreed@umich.edu (M.P.R.); 2 The Michigan Department of Transportation, Lansing, MI 48909, USA

**Keywords:** light detection and ranging, LiDAR, pavement marking detection, pavement marking materials, detectability score, detectability degradation

## Abstract

Recent advancements in vehicle automation and driver-assistance systems that detect pavement markings has increased the importance of the detectability of pavement markings through various sensor modalities across weather and road conditions. Among the sensing techniques, light detection and ranging (LiDAR) sensors have become popular for vehicle-automation applications. This study used low-cost mobile multi-beam LiDAR to assess the performance of several types of pavement marking materials installed on a limited-access highway in various conditions, and quantified the degradation in detection performance over three years. Four marking materials, HPS-8, polyurea, cold plastic, and sprayable thermoplastic, were analyzed in the current study. LiDAR reflectivity data extracted from a total of 210 passes through the test sections were analyzed. A new detectability score based on LiDAR intensity data was proposed to quantify the marking detectability. The results showed that the pavement marking detectability varied across the material types over the years. The results provide guidance for selecting materials and developing maintenance schedules when marking detectability by LiDAR is a concern.

## 1. Introduction

Various sensing technologies have been used to detect lane markings and road signs. Systems based on optical cameras have been most commonly used, and advances in machine learning techniques have enabled these machine vision-based detection systems to become increasingly reliable [1,2,3,4]. However, these vision-based systems are dependent on light conditions and sensitive to shadows and illumination noise. In contrast, light detection and ranging (LiDAR) sensors provide a 3D representation of the environment with an accuracy of a few centimeters and a strong robustness to light conditions [5,6,7].

Numerous pavement marking materials have been developed to balance day and night visibility, durability, and robustness to road conditions. For night-time visibility, the most important performance metric is the material’s optical retroreflectivity. Optical retroreflection refers to the fraction of incident light reflected back to the source light. Glass beads, for example, are now widely used in pavement markings for enhanced optical retroreflectivity, especially at night, when the primary illumination is provided by vehicle headlamps [8]. Because the glass beads do not perform as well when the road surface is wet, wet-reflective technology combines glass beads and ceramic elements to maintain a higher level of visibility across road conditions. To reduce degradation due to tire wear, pavement markings can be recessed into the road surfaces, although this tends to reduce wet performance as the recess retains water.

The majority of prior research on pavement markings has focused on detectability for human vision or optical cameras; relatively few studies have investigated the usability of the LiDAR sensing systems to detect pavement markings in real-world uses. For example, Olsen et al. [9] reported that a mobile LiDAR sensor can be effectively used for maintenance and asset-management purposes by evaluating the retroreflectivity of the extensive highway network. Hata and Wolf [10] used an Otsu thresholding method to segment road markings from asphalt in the LiDAR point-cloud data and presented a road-marking detector to recognize the types of road markings, i.e., crosswalk, dashed line, continuous line. Ghallabi et al. [11] used multilayer LiDAR data to detect lane markings using the Hough transform technique for localization within a HD map. Lee et al. [12] proposed a fusion detection system that utilizes an LiDAR-based lane marking detection technique along with a calibrated around-view-monitor (AVM) system to improve the performance in map-matching localization of a vehicle. Yang et al. [13] proposed a marking detection algorithm using a low-cost mobile multi-beam LiDAR. A series of filters such as nonroad point filter, extended moving fitting window filter, density-based adaptive window median filter and a segmentation-based filter were applied to extract road marking data from a noisy LiDAR point cloud. However, quantitative analysis on the marking detectability of pavement markings for LiDAR has been hampered by the lack of a standardized metric measuring the marking detectability in terms of LiDAR reflective intensity.

The overall goal of this pilot study was to gather LiDAR data for a variety of lane marking materials over several years to evaluate marking-performance degradations for LiDAR intensity-based marking detection systems. Documenting the pavement marking performance over time required the development of a marking detectability score in terms of LiDAR reflective intensity.

## 2. Materials and Methods

### 2.1. Hardware System

**Figure 1:**
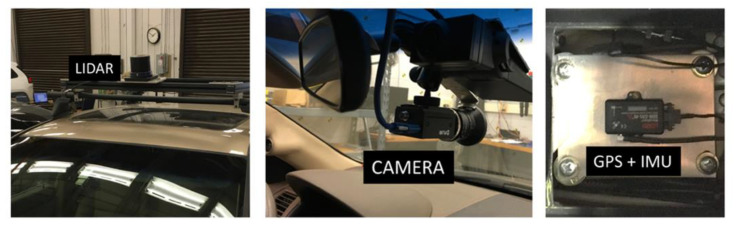
A mid-size sedan was used as a test vehicle, equipped with an LiDAR (Quanergy M8), Camera (Flir Grasshopper), and GPS+IMU device (Load).

A mid-size sedan (Honda Accord) was equipped with a 905 nm LiDAR (Quanergy M8) along with a high-fidelity camera and IMU+GPS sensor to record LiDAR data as well as the geolocation of the vehicle. Figure 1 shows the placements of the equipped sensors and the camera on the vehicle.

**Figure 2:**
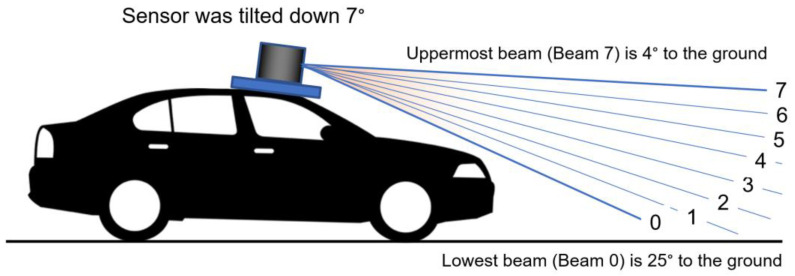
Schematic of LiDAR installation with an angled view for maximizing the frontal road scanning. The LiDAR has 8 sensing layers, from Beam 0 to Beam 7.

The M8 sensor has eight laser beams providing a 360° horizontal and 20° vertical field of view [14,15]. The measurement technique used in this sensor is time-of-flight (TOF) with a frame rate of 5 Hz to 20 Hz. In default mode, the M8 sensor spins at 10 Hz, and the laser fires at a constant rate of 53,828 Hz. To avoid any interference among the lasers, the lasers are fired in the sequence 0, 4, 2, 6, 1, 5, 3, and 7, where the Beam 0 is the lowest, downward-looking plane, and the Beam 7 is the highest as shown in Figure 2. The sensor returns angle, distance, intensity, and a synchronized timestamp for each sample. The intensity is stored as an unsigned 8-bit byte value where 0 is the lowest intensity, and 255 is the highest intensity. Note that this is not directly interpretable in the typical units of retroreflectivity but rather should be considered a relative measure. The sensor was positioned at the top of the roof of the test vehicle and tilted down 7 degrees to maximize the frontal road scanning as shown in Figure 2.

### 2.2. Software System

**Figure 3:**
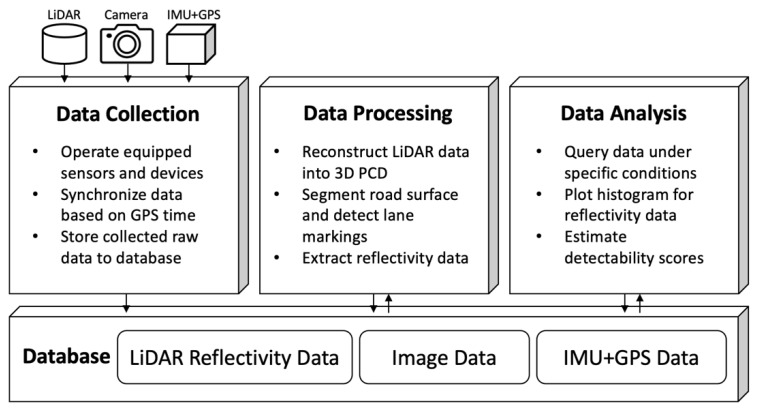
Overview of the lane-marking evaluation system.

A software system was developed to collect and analyze the lane-marking data obtained from all the vehicle sensors. The system consists of a database and three programs for data collection, processing, and analysis. Figure 3 shows the overall structure of the system.

The data collection program operates all the sensors and records heterogeneous data from the sensors in real-time using application programming interfaces (APIs) provided by the sensor manufacturers. The program captures color-image data from the camera and also records the LiDAR’s reflectivity data with GPS data by capturing the network traffic between the computer and the LiDAR. All the recorded raw data are stored in the database. The LiDAR data packets along with GPS data take about 1 MB/s on average. The camera returns 10 frames per second, and each raw image takes 4 MB.

**Figure 4:**
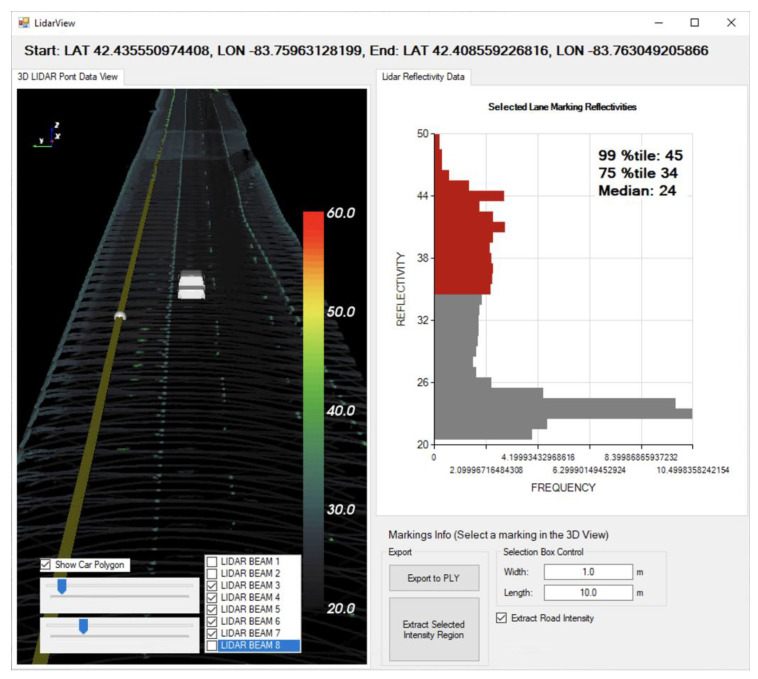
The user interface of the 3D LiDAR data viewer. LiDAR points are visualized as 3D point-cloud data, and the intensities are colorized.

The data processing program was developed to explore the database and visualize the processed LiDAR data, e.g., return intensities, within a specified geographic. Figure 4 shows a visualization module of the program that reconstructs the input LiDAR data into 3D point-cloud data with colorized intensities. Generally, the raw data obtained from a LiDAR sensor contain time-of-flight (distance) information with intensity data from each emitted light instance. This distance information needs to be converted as 3D point data based on the rotational position of the ray. In this module, every LiDAR point that has a distance to the object and intensity values is reconstructed as a 3D point using an algorithm as follows:

1. Rotational angles {*rotX*, *rotY*} of the beam for the input point is given by
  *rotX* = *cos*(*v*), *rotY* = *sin*(*v*),
  where *v* = (*p* + 0.5*v*)/*v* × 2π^2^,
  *p* is position in 1/10,400 rotation,
  *v* is the number of rotations (10,400).
2. The 3D coordinate {*x*, *y*, *z*} of the point is computed as
  *x* = *rotX* × cos(*rad*) × *dist*,
  *y* = *rotY* × cos(*rad*) × *dist*,
  *z* = *sin*(*rad*) × *dist*,
  where *rad* is the radian angle of the beam with respect to the axis of the sensor, and *dist* is the measured distance of the beam.

The converted points are then transformed based on the geolocation and vehicle rotational acceleration information as well as the sensor tilt angle. To visualize the intensities, we applied a heatmap colorization for each converted point. Additionally, the points that are 50 cm above or below the road surface level were considered to be noise and eliminated, and all the other points were projected to the surface level for better visualization. An example of reconstructed LiDAR points is shown in the left panel in Figure 4.

A semi-automated gradient-based marking detection algorithm was developed in the data processing program to extract the intensity values from a single lane marking. Once a point on a lane marking of interest is manually selected (the seed point), the program detects points related to the selected marking. Neighboring points to the seed point within a radius of 0.5 m are selected using the k-D tree technique that is a fast closest-point searching method using a space-partitioning data structure (Bentley 1975). It works by recursively partitioning the points along different axes. To find the nearest neighbor to a query point, the tree is traversed while comparing distances and pruning subtrees that cannot contain a closer point. This process continues until the entire tree is explored, resulting in the nearest neighbor. In this study, we used *VTK* v5.8 (http://www.vtk.org, accessed on 20 June 2023) for k-D tree searching. The region is divided into 180 sections of angles, and the gradients are estimated along the centerline within each section, and two opposite sections that have the minimal gradients are selected as the local lane marking direction. The farthest point from the seed point in each selected section is considered as the next seed point, and a marking line can be determined along with the seed points by iterating the steps mentioned above. Finally, points that are within 0.1 m from the marking line connecting the seed points are selected as lane-marking points.

### 2.3. Marking Reflectivity Data Analysis

Prior to the LiDAR intensity analysis, the noise due to measurement angle and the road surface was minimized from all the intensity data. The LiDAR sensor uses eight laser beams in different vertical angles to scan the surroundings, as mentioned in a previous section. Since the test vehicle’s hood occludes part of the scan path of the lowest beam and the highest beam barely captures the road, we eliminated the first and the 8th beams’ intensities. Additionally, the marking intensity data contains a wide range of road-surface intensities, in part because we extracted marking data with a 200 mm wide band around the lane marking to ensure the full marking width was captured (the widest marking width is 150 mm). The road-surface data alongside the marking line were also extracted along with the marking intensity data, and the upper edge of the distribution of the road intensities was used as the lower boundary for defining lane marking intensities.

The intensity distributions measured from the road surface without any markings were analyzed along with the marking intensities. Except during wet surface (rainy) conditions, the unmarked pavement produced intensities in the range from 0 to 30, and the 99th-percentile value was 27. Thus, we used the intensity of 29 as the upper boundary to filter the road-surface intensities from all the marking data. That is, intensity values of 30 and higher were considered to represent data from the lane markings. Note that the percentage of intensities from points on the pavement that were not lane markings rarely exceeded the chosen threshold (<1% of non-marking points) and effects on the final detectability scores were neglectable. Importantly, these false positives were equally likely regardless of the marking type and quality, and hence do not affect the ability to differentiate among different marking materials or to track their degradation.

**Figure 5:**
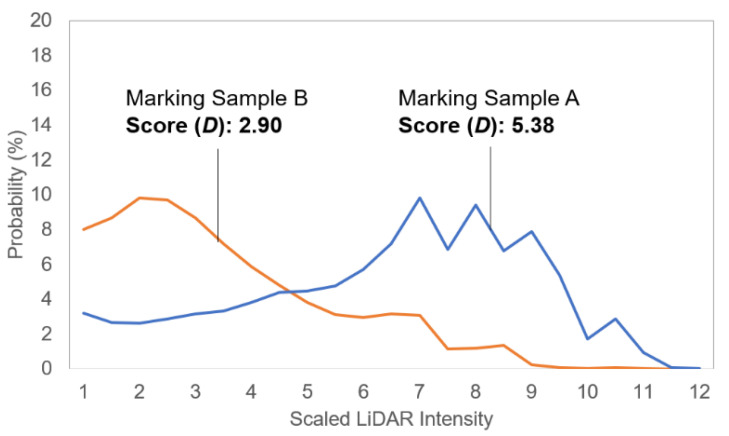
Intensity distributions of two sampled markings.

In general, the lane-marking intensities fall into a range of 30 to 50, while the full range of the intensity for the M8 LiDAR sensor is 0 to 255 (8 bits, i.e., one byte). Markings are typically detected based on the retroreflectivity discrepancy between the road and markings, so higher intensity values represent greater detectability. Figure 5 compares the processed intensity distributions of two-lane markings. Both markings have similar ranges of intensity distribution (Marking A: 27 to 50, Marking B: 27 to 49), but marking A outperforms marking B in terms of detectability due to the greater percentage of points at higher intensity values, indicating that the marking is effectively “brighter” to the sensor. Thus, the detectability performance of a marking should be assessed by considering both the intensity range and the proportional amount of higher intensity values.

A detectability score was computed from the distribution as follows. Intensities of 29 and below were discarded as background road points, and the frequencies of points with intensities of 30 and higher were computed. Each intensity frequency was multiplied by the integer intensity value over 29 and summed to obtain the detectability score. Symbolically,

(1) $$D=\sum_{i=30}^{50}S\left(V_{i}-29\right)F_{i},$$

where *V_i_* is the *i*th intensity value (from 30 to 50), *S* is a scale factor to make the score range from 1 to 11 (*S* = 0.5), and *F_i_* is the frequency of the *i*th intensity. Note that the score is dimensionless and relative, and the current score range was arbitrarily chosen to facilitate comparisons.

The scores for the sampled marking A and B were 5.38 and 2.90, respectively. This can be interpreted as marking A having about 1.8 times higher detectability.

### 2.4. Experimental Design

The Michigan Department of Transportation installed new pavement markings on US-23 test sections (~24 km in each north/south bound) with four different materials: Polyurea, HPS-8, Cold plastic, and thermal spray. These materials also had variations in geometries, widths, colors, and wet-reflective properties.

Polyurea (3M Liquid Pavement Marking Series 5000, 3M Maplewood, Minnesota, MN, USA) is a high-performing plural component material that mixes more than one compound to increase durability, color retention, and reflectivity (PK Contracting). Sprayable thermoplastic (Crown Technology, LLC) is a granular/powdered material that is applied by melting it at >400 degrees Fahrenheit to become a liquid, and which hardens as it cools. The thermal spray is 40 Mil sprayable thermoplastic, a non-durable formulation for easy construction. This material provides high adhesion, retroreflectivity, and color retention (Crown Technology, LLC). A HPS-8 integrated multipolymer (Ennis-Flint) is a hybrid of extruded thermoplastic and highly durable methyl methacrylate (MMA). This HPS-8 has a higher long-term durability and retroreflectivity than thermoplastic. Also, it is resistant to snowplow damage (Ennis-Flint). Overlay cold plastic (OVCP) (3M™ Stamark™ High-Performance Pavement Marking Tape Series 380AW) is a pre-formed tape that is applied by tamping with a rolling device to bond with the pavement surface.

**Figure 6:**
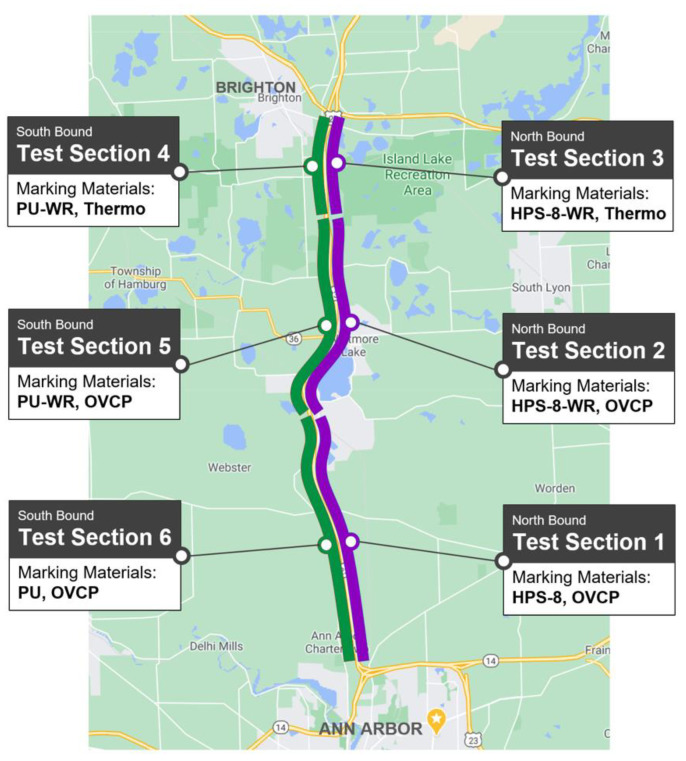
Lane-marking performance test sections on US-23 (purple: north bound, green: south bound) and installed pavement marking materials, including polyurea (PU), overlay cold plastic (OVCP), thermal spray (Thermo), and HPS-8.

On the US-23, this material was installed in the pavement marking on test sections 1, 2, 5, and 6 with the recessed-marking method. Figure 6 shows the test sections on a map, and Appendix A summarizes the matrix of pavement-marking materials and properties for each test section.

For the data collection, we collected the data from the US-23 corridor approximately one to three times per week over about three years (2018 to 2020) under various weather and road conditions, such as sunny (73%), cloudy (21%), and rainy/snowy conditions (6%) in both daytime (89%) and night (11%). Note that extreme weather conditions such as hard raining and snowing conditions were excluded for safety.

## 3. Results

A total of 210 drives on US-23 were performed, collecting data both northbound and southbound, for a total of 4719 km. The data were mostly collected in clear, dry weather (73%), with the remaining conditions classified as either cloudy and dry (21%) or rainy/snowy (6%). Exactly 89% of the data were collected in the daytime.

**Figure 7:**
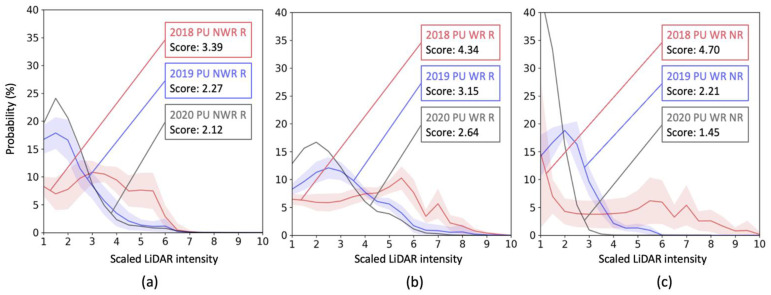
Intensity changes of polyurea (PU) markings: (**a**) recessed and non-wet-reflective markings, (**b**) recessed and wet-reflective markings, and (**c**) non-recessed and wet-reflective markings. Vertical and horizontal axes represent frequency (%) and scaled intensity value, respectively. Colored bands show ±1 standard deviation range. No band is shown for 2020 due to the small number of samples.

Figure 7 shows longitudinal changes over three years in intensity distributions of the polyurea markings. All the data under the same conditions, such as material type, measurement angle, and weather condition, were gathered for each year (2018: red, 2019: blue, 2020: black). The highlighted areas in each color show ±1 standard deviations of the data, and solid lines represent the mean intensity distribution. Note that only a few drives were performed in 2020, so standard deviations were not computed.

For non-wet-reflective and recessed polyurea markings in test section 6, the detectability deterioration appears more significant for the polyurea when a marking is newer. Over the first year (2018–2019), the detectability score was degraded by 1.12, while the second year (2019–2020) had a relatively small score reduction of 0.15. For wet-reflective and recessed polyurea markings in test section 5, similarly, the first year had a larger decreasing rate in detectability score than the second year for the non-wet-reflective polyurea. In the first two years, the score decreased by 1.19, while the reduction in the score was 0.51 between the second and third years. For wet-reflective polyurea markings installed on a non-recessed surface, the material showed the steepest detectability degradation in a year, as expected. In the first year, the score dropped by 2.49. This is a 2.1-times larger decrease rate than the score drop for the same material on a recessed surface. In the second year, the intensity decrease rate was also larger for the non-recessed polyurea markings. The detectability score dropped by 0.76, compared to the score decrease (0.51) for the recessed polyurea marking, but the difference was not bigger than the first two years.

**Figure 8:**
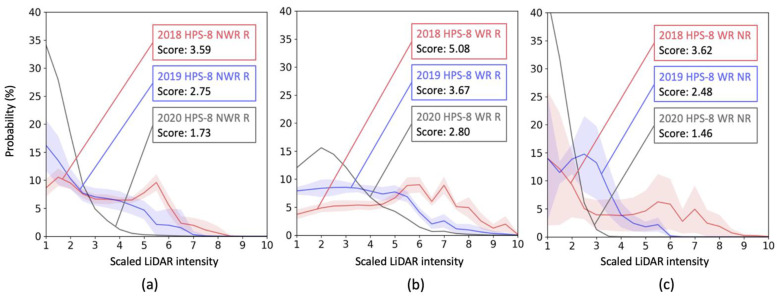
Intensity changes of HPS-8 markings: (**a**) Recessed and non-wet-reflective markings, (**b**) recessed and wet-reflective markings, and (**c**) non-recessed and wet-reflective markings.

Figure 8 shows longitudinal changes over three years in intensity distributions of the HPS-8 markings installed in the test sections 1 to 3. Figure 8a shows intensity changes for the non-wet-reflective and recessed HPS-8 markings. In the first year, the detectability score dropped from 3.59 to 2.75 (−0.84), while the score decreased to 1.73 (−1.02) in the second year. Compared to the same type of polyurea markings, the HPS-8 marking score decreased more consistently over the years. Figure 8b compares the intensity distributions of wet-reflective markings. Similarly, the material showed relatively more consistent score-degradation rates (−1.41 and −0.87 over three years) compared to the wet-reflective polyurea. Figure 8c shows the intensities of wet-reflective markings installed on a non-recessed surface. Similarly, the non-recessed markings’ detectability was degraded constantly by 1.14 in 2018–2019, and by 1.02 in 2019–2020.

**Figure 9:**
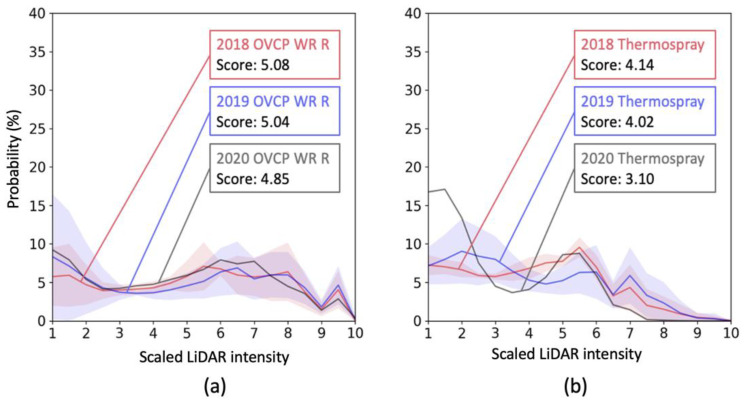
Intensity changes of (**a**) OVCP and (**b**) bThermospray markings over the years.

Figure 9a shows the intensity distributions of wet-reflective OVCP markings. The OVCP markings were installed throughout the test sections except section 3 and section 4 as dashed markings in between solid markings. The OVCP shows the best reliability among the materials over three years. The detectability scores remained almost the same level, around 5.0 through 2018 to 2020.

Figure 9b shows the intensity data of the thermal spray markings. The markings are dashed markings in test sections 3 and 4 and installed on a non-recessed surface. Again, compared to the solid lane markings, the thermal-spray markings showed a relatively reliable performance over three years but less reliability than OVCP. The detectability score dropped from 4.14 in 2018 to 3.10 in 2020, which is a bigger degradation than the OVCP dashed markings but better performance than the solid lane markings in the same test sections.

**Figure 10:**
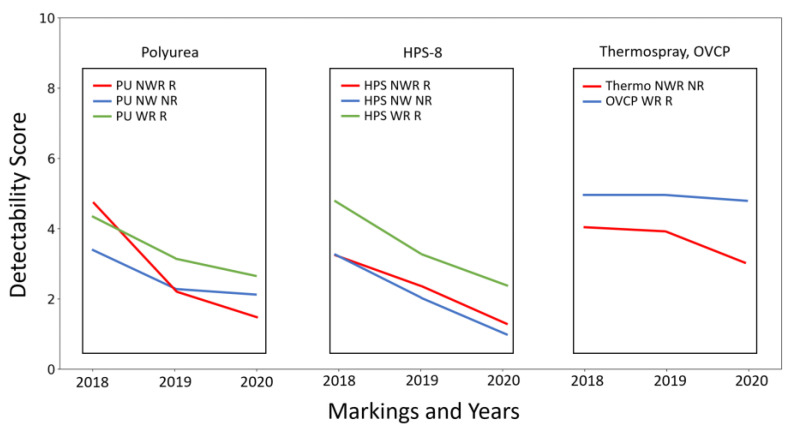
Comparisons of time effect on pavement markings over three years.

Figure 10 summarizes the detectability score decreases over three years for all the lane markings analyzed in the current study. The magnitudes of these decreases can be compared with the differences in materials when newly installed. For the recessed solid lane-marking type, the polyurea material, which is the most used marking material in Michigan, had a score decrease rate of 0.74 per year on average, while the HPS-8 had a score change of 1.04 per year on average. The decrease slowed over time for the polyurea markings, although the HPS-8 marking had a relatively consistent decrease rate. As expected, the scores of the solid lane markings on a non-recessed surface showed more rapid degradation: −0.76 and −1.02 for polyurea and HPS-8, respectively. Thermospray and OVCP (dashed lines) outperformed the solid markings with a higher score and a reliable performance.

## 4. Discussion

This study quantified the pavement-marking detectability for LiDAR sensors based on the analysis of intensity data and quantitatively analyzed the effect of time on the marking detectability for LiDAR sensors. Although LiDAR retroreflectivities have been analyzed in previous studies, a common fundamental limitation is that measurements are taken at only a few, discrete locations. However, we have collected various pavement marking materials over three years and analyzed the real degradation in marking detectability for LiDAR sensors, which will be very practical data for utilizing LiDAR in real-world uses.

The results showed that the new detectability score can reasonably represent the LiDAR intensity distribution of a pavement marking. The score quantifies both the intensity range and the proportion of higher-intensity returns, which are the key factors to determine the detectability of markings. The score is limited in that it applies only to the current sensor and dataset and the absolute values cannot be interpreted with respect to standard measures of intensity. Rather, the score was developed to provide useful evaluation of material differences and longitudinal effects.

The results also showed that the time effect on detectability degradation can be quantified using the scores. The magnitudes of these degradations can be compared with the differences in materials when newly installed. For the recessed solid lane-marking type, the polyurea material, which is the most used marking material in Michigan, had a score decrease rate of 0.74 per year on average, while the HPS-8 had a score change of 1.04 per year on average. The decrease slowed over time for the polyurea markings, although the HPS-8 marking had a relatively consistent decrease rate. As expected, the scores of the solid lane markings on a non-recessed surface showed more rapid degradation: −0.76 and −1.02 for polyurea and HPS-8, respectively. Thermospray and OVCP (dashed lines) outperformed the solid markings with a higher score and a reliable performance.

The limitations of the current method is that the reflective intensity data would likely be different with a different type of sensor, based on the type of light source, scanning technology, and sensor intrinsic parameters. Also, a different LiDAR positioning on a vehicle and different reading scenarios, such as occlusion or incompleteness, could affect the reflective intensity. We plan to collect more data under a wide range of scenarios and conditions to overcome these limitations. Also, further calibration on collected intensity data, e.g., the deterministic calibration [16], could be used to address this issue.

Almost all the pavement markings we tested showed good detectability with fair separations from the road surface in terms of LiDAR retroreflectivity. Both polyurea and HPS-8 pavement markings (solid lane markings) showed intensity degradation over three years. Based on the observed trends, we can expect that the marking intensities will fall into the road surface intensity range (score under zero) after some period. The wet reflective technology had a slightly better score than the others but was not effective for LiDAR when the surface was fully wet. The system used in the current study could not extract the marking intensity data for the wet pavement due to a lack of differentiation in intensity between the marking and the adjacent pavement. In contrast, the markings were still clearly visible to the eyes. This indicates that a hybrid approach that utilizes a LiDAR system along with a vision-based marking detection system would provide more robust marking detection performance than a LiDAR-only system.

## 5. Conclusions

This pilot study examined the longitudinal degradation of marking detectability using LiDAR sensors. Four marking materials were tested, and their performance was evaluated over three years. We proposed a new metric to quantify marking detectability based on LiDAR intensity patterns. The results demonstrated variations in detectability, durability, reliability, and degradation rates among the tested materials.

Solid markings showed good durability, with degradation rates increasing due to traffic. Recessed marking technology effectively maintained marking performance for LiDAR, while wet reflective materials performed better under certain conditions. LiDAR proved robust for marking capture regardless of daytime or nighttime but was sensitive to surface wetness.

To our knowledge, this is the first longitudinal study investigating LiDAR intensity trends of pavement markings over a long period of time in real-world use. Results will be useful for future pavement-marking planning and material selections.

This study provides valuable insights for future pavement-marking planning and material selections. However, further research is needed to understand the nonlinearity of degradation rates, standardize intensity readings across different devices, analyze the effects of independent variables, assess new pavement-marking technologies, and explore manufacturers’ perspectives on using LiDAR for marking detection. Additionally, analyzing road signs and markings on urban roads using our method would be beneficial.

## Data Availability

Not applicable.

## Appendix A

**Table A1:** Matrix of marking materials and properties for test sections.

Bound	Test Section	Location	Material	Color	Geometry	Wet Reflective	Recessed
North	1	(42.3305, −83.7429)–(42.4072, −83.7625)	HPS-8	Y	Solid	No	Yes
			HPS-8	Y	Solid	No	Yes
			OVCP	W	Dashed	Yes	Yes
			HPS-8	W	Solid/Dashed	No	Yes
	2	(42.4072, −83.7625)–(42.4617, −83.7548)	HPS-8	Y	Solid	Yes	Yes
			HPS-8	Y	Solid	Yes	Yes
			OVCP	W	Dashed	Yes	Yes
			HPS-8	W	Solid/Dashed	Yes	Yes
	3	(42.4617, −83.7548)–(42.5198, −83.7551)	HPS-8	Y	Solid	Yes	No
			Thermospray	W	Dashed	No	No
			HPS-8	W	Solid/Dashed	No	No
South	4	(42.5198, −83.7550)–(42.4364, −83.7585)	Polyurea	Y	Solid	Yes	No
			Thermospray	W	Dashed	No	No
			Polyurea	W	Solid/Dashed	No	No
	5	(42.4364, −83.7585)–(42.4072, −83.7625)	Polyurea	Y	Solid	Yes	Yes
			Polyurea	Y	Solid	Yes	Yes
			OVCP	W	Dashed	Yes	Yes
			Polyurea	W	Solid/Dashed	Yes	Yes
	6	(42.4079, −83.7625)–(42.3305, −83.7429)	Polyurea	Y	Solid	No	Yes
			Polyurea	Y	Solid	No	Yes
			OVCP	W	Dashed	Yes	Yes
			Polyurea	W	Solid/Dashed	No	Yes
